# Development and validation of a radiomics-based nomogram for the preoperative prediction of microsatellite instability in colorectal cancer

**DOI:** 10.1186/s12885-022-09584-3

**Published:** 2022-05-09

**Authors:** Mingliang Ying, Jiangfeng Pan, Guanghong Lu, Shaobin Zhou, Jianfei Fu, Qinghua Wang, Lixia Wang, Bin Hu, Yuguo Wei, Junkang Shen

**Affiliations:** 1 Department of Radiology, The Second Affiliated Hospital of Soochow University, No.1055 Sanxiang Road, Gusu District, Suzhou, 215004 Jiangsu China; 2 Department of Radiology, Jinhua Hospital of Zhejiang University: Jinhua Municipal Central Hospital, No. 351 Mingyue Road, Jinhua, Zhejiang, China; 3 Department of Oncology, Jinhua Hospital of Zhejiang University: Jinhua Municipal Central Hospital, No. 351 Mingyue Road, Jinhua, Zhejiang, China; 4 Department of Pathology, Jinhua Hospital of Zhejiang University: Jinhua Municipal Central Hospital, No. 351 Mingyue Road, Jinhua, Zhejiang, China; 5 Precision Health Institution, GE Healthcare, Xihu District, Hangzhou, China; 6 Institute of Radiation Oncology Therapeutics of Soochow University, Suzhou, 215004 China

**Keywords:** Microsatellite instability, Colorectal cancer, Radiomics, Computed tomography

## Abstract

**Background:**

Preoperative prediction of microsatellite instability (MSI) status in colorectal cancer (CRC) patients is of great significance for clinicians to perform further treatment strategies and prognostic evaluation. Our aims were to develop and validate a non-invasive, cost-effective reproducible and individualized clinic-radiomics nomogram method for preoperative MSI status prediction based on contrast-enhanced CT (CECT)images.

**Methods:**

A total of 76 MSI CRC patients and 200 microsatellite stability (MSS) CRC patients with pathologically confirmed (194 in the training set and 82 in the validation set) were identified and enrolled in our retrospective study. We included six significant clinical risk factors and four qualitative imaging data extracted from CECT images to build the clinics model. We applied the intra-and inter-class correlation coefficient (ICC), minimal-redundancy-maximal-relevance (mRMR) and the least absolute shrinkage and selection operator (LASSO) for feature reduction and selection. The selected independent prediction clinical risk factors, qualitative imaging data and radiomics features were performed to develop a predictive nomogram model for MSI status on the basis of multivariable logistic regression by tenfold cross-validation. The area under the receiver operating characteristic (ROC) curve (AUC), calibration plots and Hosmer-Lemeshow test were performed to assess the nomogram model. Finally, decision curve analysis (DCA) was performed to determine the clinical utility of the nomogram model by quantifying the net benefits of threshold probabilities.

**Results:**

Twelve top-ranked radiomics features, three clinical risk factors (location, WBC and histological grade) and CT-reported IFS were finally selected to construct the radiomics, clinics and combined clinic-radiomics nomogram model. The clinic-radiomics nomogram model with the highest AUC value of 0.87 (95% CI, 0.81–0.93) and 0.90 (95% CI, 0.83–0.96), as well as good calibration and clinical utility observed using the calibration plots and DCA in the training and validation sets respectively, was regarded as the candidate model for identification of MSI status in CRC patients.

**Conclusion:**

The proposed clinic-radiomics nomogram model with a combination of clinical risk factors, qualitative imaging data and radiomics features can potentially be effective in the individualized preoperative prediction of MSI status in CRC patients and may help performing further treatment strategies.

**Supplementary Information:**

The online version contains supplementary material available at 10.1186/s12885-022-09584-3.

## Background

Colorectal cancer (CRC) is the third most commonly diagnosed malignant tumor and remains the second leading cause of mortality from cancer worldwide [1]. Mismatch repair deficiency (dMMR) or microsatellite instability (MSI) is observed in approximately 15% of CRC, and MSI is a hypermutable phenotype caused by replication errors in DNA mismatch repair [2]. MSI or dMMR identifies a unique subset of CRC with favorable prognostic, therapeutic and negative predictive relevance [3]. MSI CRC patients can avoid the side effects of fluorouracil-based adjuvant chemotherapy, which has limited value in CRC with MSI status, while they can show a likely better prognosis and clinical benefit from immune checkpoint inhibitors in late-stage CRC patients [4]. Furthermore, MSI and dMMR are associated with Lynch syndrome via a unique pathway in carcinogenesis, representing the most common inherited disease leading to CRC [5]. Because of the dramatic clinical demand for MSI/dMMR status, MSI/dMMR status testing is recommended for all CRC patients by international guidelines such as the National Comprehensive Cancer Network (NCCN) guidelines [4] and the European Society for Medical Oncology (ESMO) guidelines [3]. MSI/dMMR detection relies on pathological tissues via polymerase chain reaction (PCR), immunohistochemistry (IHC) or genetic analyses [6]. However, pathological CRC tissues are preoperatively obtained by invasive colonoscopy biopsy requiring a group of gastroenterologists, anesthesiologists, and nurses for the procedure, which can be costly and time-consuming. Colonoscopy biopsy can only obtain a small proportion of CRC tissue samples, which may not meet the minimum quantity criteria for these advanced biological tests [7]. Furthermore, these advanced biological tests may be available only in tertiary care centers, which prevents identification of MSI status in many CRC patients. Thus, it is crucial to develop a noninvasive, cost-effective and preoperative method to predict MSI status, which could be useful for clinicians to perform further treatment strategies.

In routine clinical practice, computed tomography (CT) is a preferred first-line noninvasive approach that has been widely used to guide treatment planning strategies for CRC patients. Furthermore, radiomics, as an emerging technique that extracts high-throughput textural features from medical images, converts medical images into structural information and mineable data regarding the underlying pathophysiology or genetic changes, thus providing deep characterization of tumor phenotypes [8, 9]. Specifically, CT-based radiomics has demonstrated clinical value in the preoperative prediction of KRAS/BRAF mutation status in CRC patients and EGFR mutation status in lung adenocarcinoma patients [10–12]. A few scholars reported the values in the preoperative prediction of MSI status in CRC by radiomics features extracted from contrast-enhanced CT images; however, the protocol of radiomics analysis or the diagnosis efficiency was limited [13, 14], and more recent works about method for key feature selection in radiomics and radiomics workflow to create a predictive model using machine learning algorithms [15, 16]. Therefore, our retrospective study was performed to develop and validate a noninvasive, cost-effective reproducible and individualized radiomics-based nomogram method for preoperative MSI/dMMR status prediction based on contrast-enhanced CT (CECT) images.

## Methods

### Patient population

**Fig. 1:**
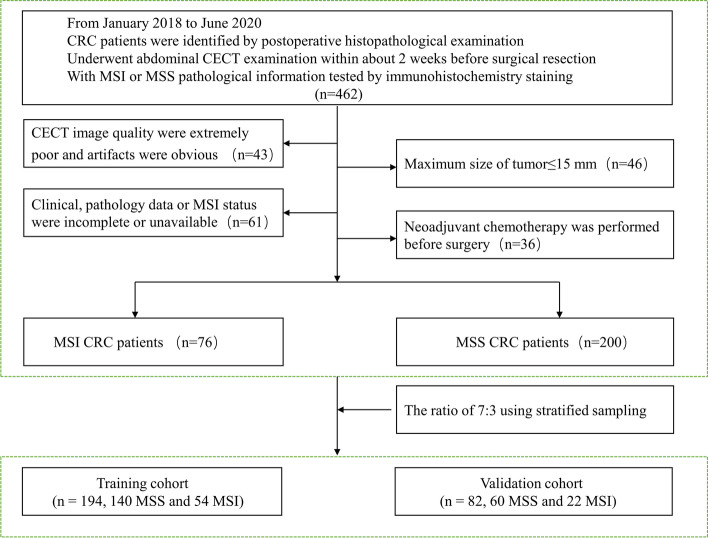
Flowchart of patient recruitment pathway

Our local institutional review board (Committee on Ethics of Biomedicine, Affiliated Jinhua Hospital, Zhejiang University School of Medicine) approved this research, and the requirement for patient informed consent was waived for this retrospective study. From January 2018 to June 2020, 462 consecutive colorectal cancer patients with pathologically confirmed disease were initially retrieved, and the patients were classified into the MSI group (*n* = 87) and microsatellite stability (MSS) group (*n* = 375). The incidence rate of MSI was 18.83% (87/462), and the prevalence of MSI was 18.83% (87/462). The inclusion criteria were as follows: (a) colorectal adenocarcinoma was identified by postoperative histopathological examination; (b) patient underwent abdominal CECT examination within approximately 2 weeks before surgical resection; and (c) pathological information on MSI or MSS tested by immunohistochemistry (IHC) was provided. The exclusion criteria were as follows: (a) CECT image quality was extremely poor, and artifacts were obvious, which could make it difficult to identify lesion delineation (*n* = 43); (b) sex, age, information on laboratory examination, pathology data or MSI status were incomplete or unavailable (*n* = 61); (c) the maximum size of tumor≤15 mm (*n* = 46); and (d) neoadjuvant chemotherapy was performed before surgery (*n* = 36). Finally, a total of 76 MSI CRC patients and 200 MSS CRC patients who met these inclusion and exclusion criteria were identified and enrolled in our study. The flowchart of the patient recruitment pathway is shown in Fig. 1.

Baseline clinical data, including patient sex, age, location of primary cancer (right colon/left colon/rectum), carcinoembryonic antigen (CEA) (normal range, 0–5 U/ml), and white blood cell count (WBC) (normal range, 3.50–9.50 × 10^9^/L), were all recorded from electronic medical records. Histological grade was directly obtained from preoperative histopathological biopsy, and MSI/dMMR status was directly obtained from histopathological and IHC reports performed after surgical resection. The CRC patients included in this study were divided into a training cohort (*n* = 194, 140 MSS and 54 MSI) and a validation cohort (*n* = 82, 60 MSS and 22 MSI) according to a ratio of 7:3 using stratified sampling to maintain the same ratio of negative to positive samples in the training and validation sets.

### Microsatellite instability status assessment

The MSI/MSS status was determined with standard IHC staining of four MMR proteins including MLH1, MSH2, MSH6, and PMS2. IHC staining was based on routinely performed postoperative formalin-fixed paraffin-embedded specimens using the standard procedure of streptavidin biotin-peroxidase. The results of IHC staining for MMR proteins were diagnosed by two expert pathologists who were blinded to the CRC patients’ available clinical and histopathological characteristics. The consistent opinions were acquired. If there were different opinions, their disagreements were resolved through negotiation. According to the results of IHC staining for four MMR proteins, CRC patients were divided into the MSS or MSI group. CRC specimens with at least one negatively stained MMR protein were subclassified into the MSI group; others with all positively stained MMR proteins were subclassified into the MSS group [5].

### CT Acquisition and image analysis

Abdominal CT scans were performed in the supine position on a 256-slice Brilliance iCT scanner (Philips Healthcare). The scan and reconstruction parameters were tube voltage, 120 kVp; tube current, 150 to 500 mA using automatic tube current modulation; collimation, 128.0 × 0.625 mm; pitch factor, 0.90 mm/rotation; slice thickness, 5 mm; increment, 5.0 mm; matrix, 512 × 512; scan field of view, 350 × 350 mm; and reconstruction kernel, standard. All scans were from the top of the liver to the pubic symphysis in the craniocaudal direction. All patients were instructed to hold their breath during the CT examination. The non-contrast abdominal CT scan was acquired first. After the nonenhanced abdominal CT scan, 80–100 mL of iodinated contrast material (iodine concentration 300 mg/mL, Omnipaque-300, GE Diagnostics) was injected at a rate of 3.0 to 3.5 mL/s during contrast-enhanced scanning. After contrast administration, contrast images were obtained at the arterial phase (25–35 s), portal venous phase (65–80 s), and delayed phase (5 min). After CECT scans, we retrieved venous phase images from the picture archiving and communication system (PACS) and advanced workstation ISP (Philips Healthcare) in the hospital for lesion segmentation and analysis.

Image analysis was performed by two senior abdominal radiologists (L.G. and P.J., with 20 and 15 years of experience in abdominal radiology, respectively), who were blinded to the clinical and histopathological information of CRC patients and conducted the evaluation together. Their disagreements were resolved by negotiation. The following qualitative imaging data extracted from CECT images were analyzed and recorded: (a) CT-reported tumor maximum size (TMS), defined as the maximum diameter of tumors on curved planar reformation images (curved line was plotted along the center of the affected bowel at a workstation ISP); (b) CT-reported T stage, determined according to the 8th AJCC staging system [17]; (c) CT-reported lymph node (LN) status, metastatic lymph node was defined as enlarged lymph node (short-axis diameter > 1 cm), circular appearance and homogeneous enhancement [18]; and (d) CT-reported inflammatory response (IFS), defined as irregular exudative manifestation from the serosal surface and/or clouding of the peritumoral fat and/or thickened mesorectal fascial reflections.

### Tumor segmentation and feature extraction

The regions of interest (ROIs) were delineated manually on the portal venous CECT images by two experienced radiologists independently via open-source ITK-SNAP software (version 3.8.0; www.itksnap.org). The ROIs were required to include the bleeding area and tumor necrosis and to exclude peri-enteric fat, adjacent air, intestinal contents and normal tissues. Both radiologists were blinded to MSI/MSS status. If there were multiple colorectal lesions, the radiologists identified the tumor according to surgical records or pathological reports. Radiologist 1 (Y.M. with 10 years of experience in abdominal radiology) and Radiologist 2 (Z.S. with 12 years of experience in abdominal radiology) performed the segmentation of 35 patients (20 MSS, 15 MSI) randomly selected from the whole study. Radiologist 1 repeated the segmentations of the above 35 patients 4 weeks later and performed ROI segmentation of the remaining patients. We evaluated the reproducibility and reliability of feature extraction by the intra- and interclass correlation coefficient (ICC). An ICC greater than 0.75 showed good consistency of feature extraction.

Subsequently, a total of 1037 high-throughput radiomics features for each patient were automatically extracted from Artificial Intelligent Kit software (A.K. software, GE Healthcare), which followed the reference manual by the Image Biomarker Standardization Initiative (IBSI). All features were classified into four groups: (a) First-order statistics features (*n* = 18); (b) Shape features (*n* = 14); (c) Texture features, such as gray-level co-occurrence matrix (GLCM, *n* = 24), gray-level size zone matrix (GLSZM, *n* = 16), gray-level run-length matrix (GLRLM, *n* = 16), neighboring gray tone difference matrix (NGTDM, *n* = 5), gray-level dependence matrix (GLDM, *n* = 14); (d) Laplacian of Gaussian (LoG) transform features (*n* = 186) and (e) Wavelet transform features (*n* = 744).

### Radiomics feature selection

All 1037 radiomics features were analyzed further based on the training dataset. We used a three-step feature selection procedure. First, we applied a variance threshold method (ICC) to reduce the number of features, and radiomic features with high reproducibility (ICC values>0.75) were retained from the feature set. Second, a multivariate ranking method called minimal-redundancy-maximal-relevance (mRMR) was applied to eliminate the redundant and irrelevant features on the basis of a heuristic scoring criterion, and only the top ranked 20 features were retained [19, 20]. Then, the least absolute shrinkage and selection operator (LASSO) was used to choose the most valuable subset of features from the top ranked 20 features. The regular parameter (λ) of LASSO regression was chosen when the average mean square error was minimal by tenfold cross validation. Moreover, the most valuable subset of features was utilized to calculate the radiomics signature score (Rad-score) and construct the final predictive model.

### Prediction building and evaluation of the radiomics nomogram model

To explore whether clinical factors and CT-reported CRC status had additional power in predicting MSI status, univariate logistic regression was applied to the training dataset for each potential risk factor, including clinical factors (age, sex, location of primary cancer, CEA, WBC and histological grade) and CT-reported CRC status (CT-reported TMS, CT-reported T stage, CT-reported LN status and CT-reported IFS), to choose the independent prediction risk factors. The selected independent prediction risk factors and Rad-score were used to develop a prediction model for MSI status on the basis of multivariable logistic regression. Then, the clinics-radiomics nomogram was constructed. Calibration plots and the Hosmer–Lemeshow test were applied to estimate the calibration of the clinic-radiomics nomogram model, and a nonsignificant test statistic indicated that the nomogram model predicted MSI status perfectly versus actual observed probability. The prediction performance of the radiomics model, the clinics model and the combined clinics-radiomics nomogram model was assessed by receiver operator characteristic (ROC) curve analysis by calculating the sensitivity, specificity, and accuracy of the area under the curve (AUC) in the training and validation cohorts. Decision curve analysis (DCA) was performed to determine the clinical utility of the radiomics and clinic-radiomics nomogram by quantifying the net benefits of threshold probabilities.

### Statistical analysis

**Fig. 2:**
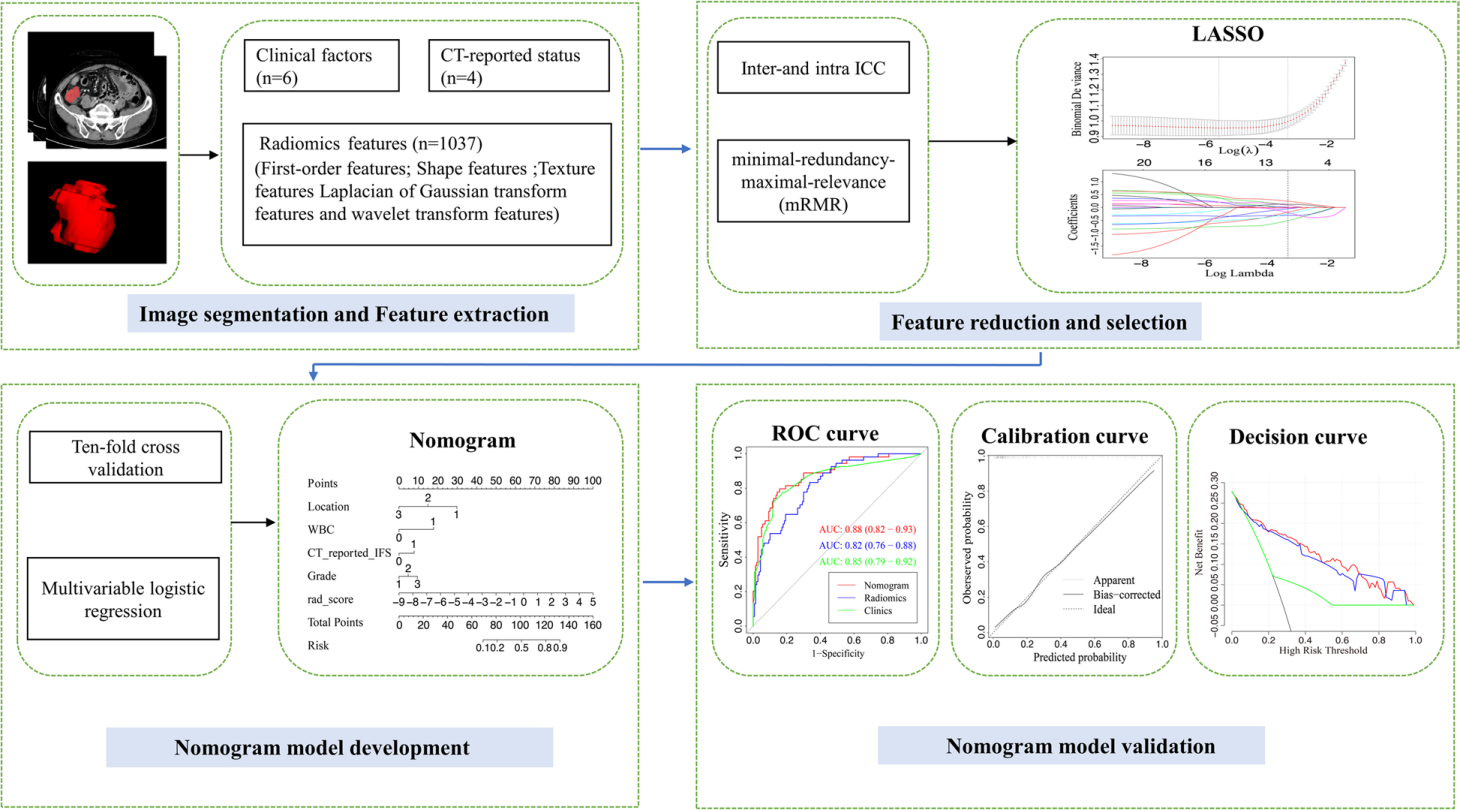
Workflow of radiomics analysis. The workflow of radiomics analysis illustrated an overview of the contrast enhanced CT imaging segmentation and feature extraction, feature reduction and selection, nomogram model development, and nomogram model validation

All statistical analyses were performed with R software, version 3.6.0 (https://www.r-project.org/). Continuous variables are presented as the mean ± standard deviation and were analyzed using either independent t tests or Mann–Whitney–Wilcoxon tests according to the distributions of the variables. Categorical variables were described as proportions and were analyzed using the chi-square test or Fisher’s exact test. A two-sided *P* < 0.05 was accepted as statistically significant. The workflow of the radiomics-based nomogram is shown in Fig. 2.

## Results

### Patient demographics and CT-reported status

The clinical factors and CT-reported status of the CRC patients in the training and validation sets are shown in Supplementary Table S1. There were no statistically significant differences detected between the training and validation sets. MSI status was significantly associated with the location of the primary tumor, histological grade, CT-reported TMS, CT-reported IFS, and Rad-score on univariate logistic analysis in both the training and validation sets, as shown in Table 1.

**Table 1:** Demographics comparison between MSI and MSS group in training and validation sets

Characteristic	Level	Training set (***n*** = 194)			Validation set (***n*** = 82)		
		MSS (***n*** = 140)	MSI (***n*** = 54)	***p***-value	MSS (***n*** = 60)	MSI (***n*** = 22)	***p***-value
**Age, mean (SD), years**		64 (11.1)	63.6 (14.1)	0.815	64.2 (12.4)	64.3 (15.9)	0.986
**gender, n (%)**	Female	55 (39.3)	26 (48.1)		24 (40.0)	17 (77.3)	
	Male	85 (60.7)	28 (51.9)	0.337	36 (60.0)	5 (22.7)	0.006
**location**, **n** (%)	Right colon	15 (10.7)	28 (51.9)		10 (16.7)	13 (59.1)	
	Left colon	23 (16.4)	16 (29.6)		5 (8.3)	7 (31.8)	
	Rectum	102 (72.9)	10 (18.5)	< 0.001	45 (75.0)	2 (9.1)	< 0.001
**CEA, n (%)**	Normal	95 (67.9)	38 (70.4)		41 (68.3)	16 (72.7)	
	Abnormal	45 (32.1)	16 (29.6)	0.868	19 (31.7)	6 (27.3)	0.910
**WBC, n (%)**	Normal	125 (89.3)	36 (66.7)		48 (80.0)	16 (72.7)	
	Abnormal	15 (10.7)	18 (33.3)	< 0.001	12 (20.0)	6 (27.3)	0.686
**CT-reported-TMS, mean (SD), cm**		4.2 (1.7)	5.8 (2.7)	< 0.001	4.3 (1.5)	6.1 (2.8)	< 0.001
**CT-reported T stage, n (%)**	T1	6 (4.3)	0 (0.0)		2 (3.3)	1 (4.5)	
	T2	9 (6.4)	2 (3.7)		4 (6.7)	2 (9.1)	
	T3	46 (32.9)	9 (16.7)		23 (38.3)	3 (13.6)	
	T4	79 (56.4)	43 (79.6)	0.021	31 (51.7)	16 (72.7)	0.209
**CT-reported LN status, n (%)**	Negative	91 (65.0)	37 (68.5)		40 (66.7)	13 (59.1)	
	Positive	49 (35.0)	17 (31.5)	0.768	20 (33.3)	9 (40.9)	0.707
**CT-reported-IFR, n (%)**	No	102 (72.9)	17 (31.5)		43 (71.7)	10 (45.5)	
	Yes	38 (27.1)	37 (68.5)	< 0.001	17 (28.3)	12 (54.5)	0.045
**Histological grade, n (%)**	Well	10 (7.1)	1 (1.9)		4 (6.7)	0 (0.0)	
	Moderately	119 (85.0)	33 (61.1)		54 (90.0)	17 (77.3)	
	Poorly	11 (7.9)	20 (37.0)	< 0.001	2 (3.3)	5 (22.7)	0.011
**Rad-score** **(median [IQR])**		−0.9[−1.6, −0.2]	0.4 [−0.4, 1.1]	< 0.001	−0.7 [−1.5, 0.0]	0.5 [−0.2, 0.9]	< 0.001

*CRC* Colorectal cancer, *CEA* Carcinoembryonic antigen, *WBC* White blood cell count, *LN* Lymph node, *MSI* Microsatellite instability, *MSS* Microsatellite stability, *TMS* Tumor maximum size, *IFR* Inflammatory response

### Inter- and intra-observer feature selection and Rad-score building

**Fig. 3:**
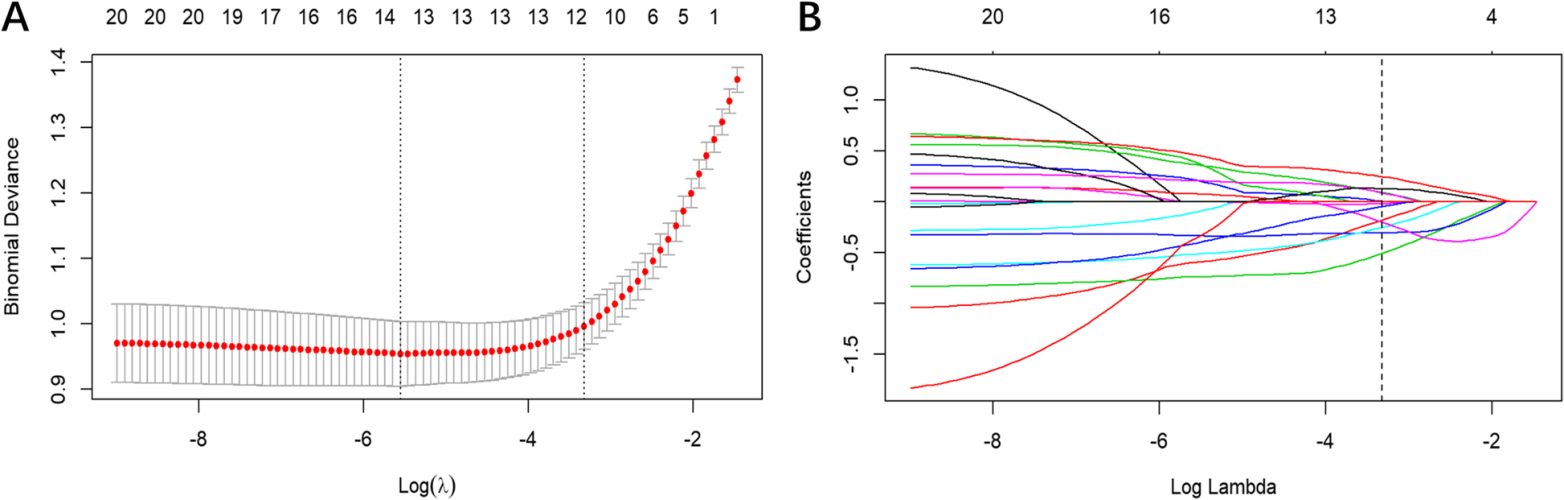
Feature selection selected by the least absolute shrinkage and selection operator (LASSO) algorithm. **A** Tuning parameter (Lambda, λ) selection in the LASSO model was optimized by 10-fold cross-validation via minimum criteria of the loss function. The dotted vertical lines in the figure and the optimal λ value of 0.03607348 were chosen corresponding to the partial likelihood estimation. **B** The vertical line was plotted at the optimal λ value, and twelve features with non-zero coefficient were selected

**Fig. 4:**
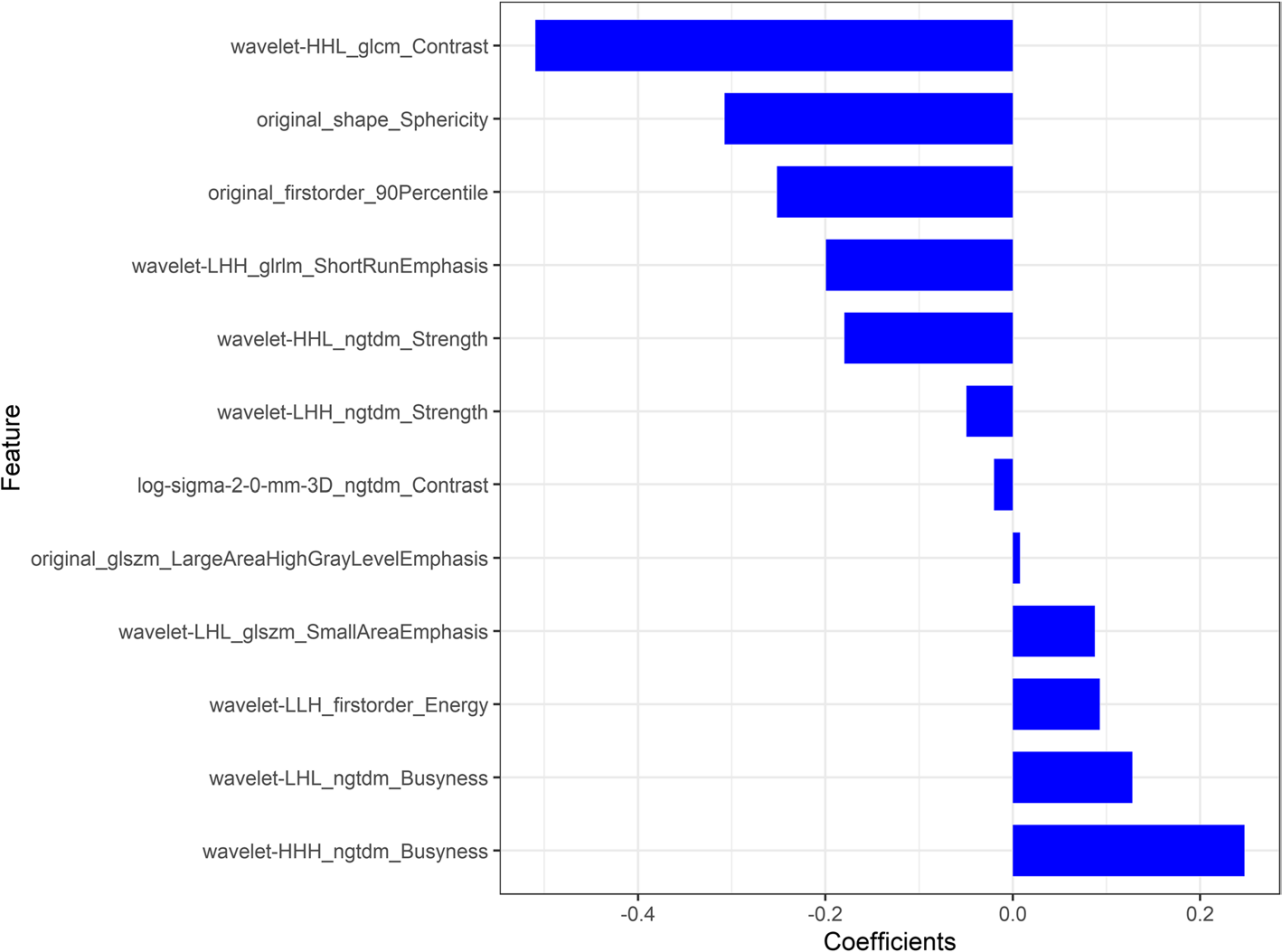
The chosen subset of radiomics features. The most twelve predictive subset of feature was chosen and the corresponding coefficients were evaluated

**Fig. 5:**
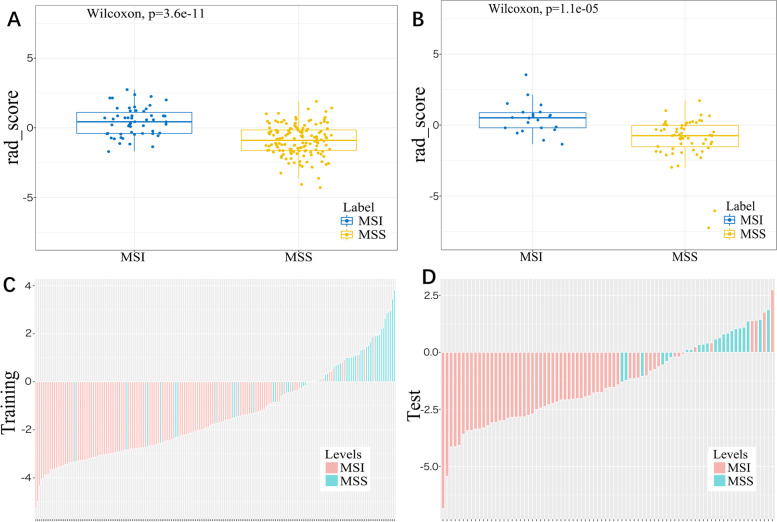
The boxplots and bar charts of Rad-score in the MSI and MSS groups. Boxplots (**A**, **B**) showed the radiomics score (Rad-score) derived from logistic regression analysis for each patient, and Bar charts (**C**, **D**) showed the bar charts of the corresponding Rad-score in the training and validation sets respectively

The radiomics features with high reproducibility (ICC values >0.75) were as follows: For intra-observer feature reproducibility and interobserver feature reliability, 820 radiomics features between the two analyses of the radiologist (P.J.) and 703 radiomics features between the second analysis of the radiologist (P.J.) and radiologist (Z.S.) were retained. Finally, 690 features were considered stable features with both interobserver reliability and intraobserver reproducibility. These 690 features acquired by radiologist (P.J.) in the first analysis were applied for further analysis. The mRMR and LASSO regression commonly applied in the regression of high-dimensional data was applied to identify the most effective predictive features for constructing the radiomic signature (Fig. 3). Finally, the most predictive subset of twelve radiomics features with nonzero coefficients was chosen to calculate the Rad-score (Fig. 4)**.** The Rad-score was calculated by summing the selected most predictive features weighted by their coefficients. The final formula of the Rad-score is shown in Supplementary Material S2. The Rad-score for each patient in the training and validation sets with regard to the MSI and MSS are shown in Fig. 5. The MSI group showed a statistically higher Rad-score than the MSS group in both the training and validation sets (*P* < 0.001) (Fig. 5).

### Development and validation of the radiomics models

**Fig. 6:**
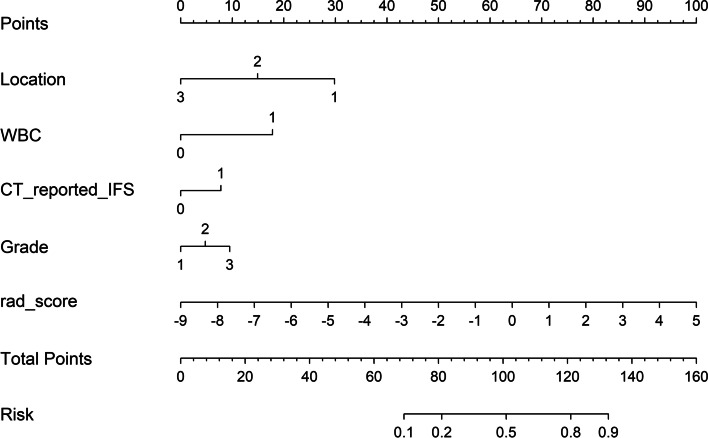
Radiomics nomogram for predicting the probability of MSI status. The Radiomics Nomogram is built based on the location, WBC, CT-reported-IFR, histological grade and the Rad-score. For location, 1 for right colon, 2 for left colon and 3 for rectum for grade. For WBC, 0 represents within the normal value used in clinics while 1 represents above the normal threshold value. For CT-reported IFR, 1 for yes and 0 for no. For histological grade, 1 for high-differentiation, 2 for middle-differentiation and 3 for low-differentiation. For Rad-score, the number represents the value of rad-score. To use, locate each variable on the axis and draw a line straight upward to the points axis to obtain the corresponding point. By summing all points and locating on the bottom line, the estimated probability of MSI status could be determined

**Fig. 7:**
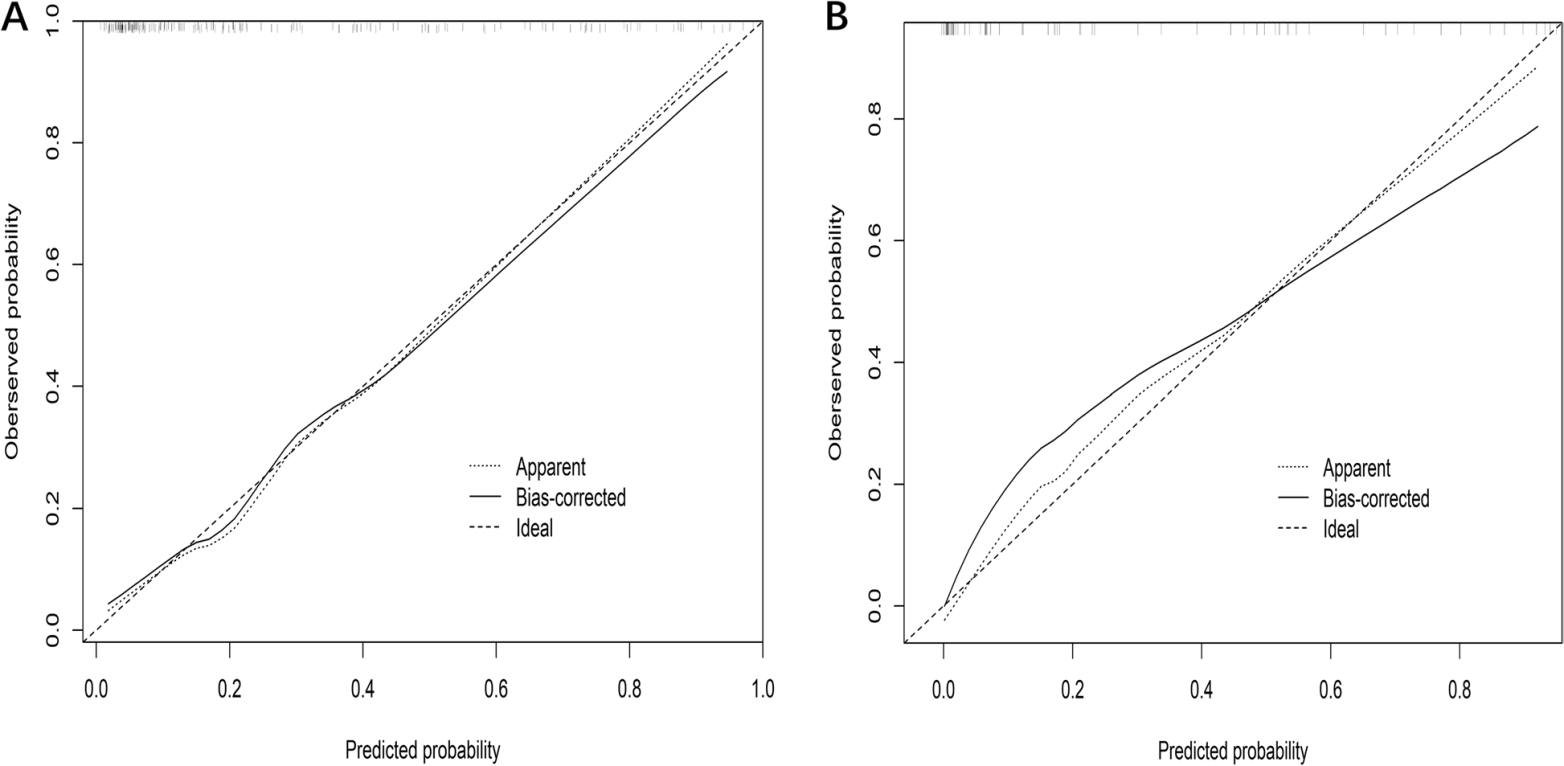
Calibration curves for radiomics based nomogram models in the training (**A**) and validation (**B**) sets. The diagonal dashed reference line indicated a perfect performance of the radiomics based nomogram by an ideal model. Solid lines indicated the performance of the radiomics based nomogram, and the diagonal dashed reference line closer alignment with solid line indicated a better performance

After multivariate logistic regression analysis, location of primary tumor (OR = 0.29 [95% confidence interval (CI), 0.18–0.48]), WBC (OR = 3.19 [95% CI, 1.13–9.03]), CT-reported IFR (OR = 3.06 [95% CI, 1.34–7.01]), and histological grade (OR = 2.42 [95% CI, 0.91–6.46]), combined with the Rad-score (OR = 1.89 [95% CI, 1.18–3.13]) were identified as independent risk predictors in the prediction model for MSI (Fig. 6). Then, we established three predictive models (radiomics, clinics and nomogram) using the above selected features. The calibration plot of the CCR nomogram model showed a statistically nonsignificant Hosmer–Lemeshow test (*P* = .417, *P* = .268) and indicated good agreement between prediction and actual observation in both the training and validation sets (Fig. 7).

**Fig. 8:**
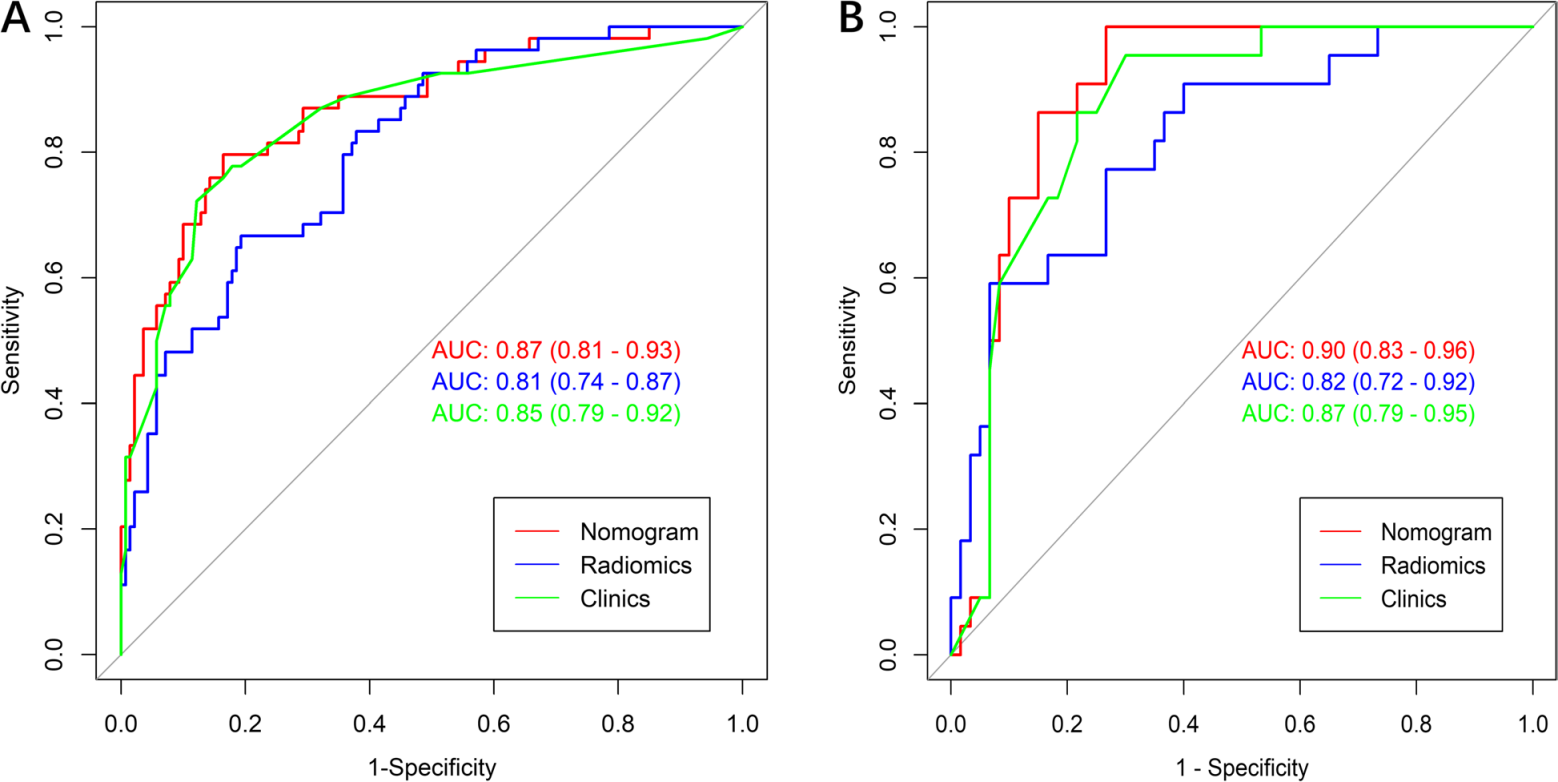
Comparing the performance in predicting the MSI status in the training (**A**) and validation (**B**) sets. Radiomics based nomogram model [area under the curve (AUC) = 0.87 and 0.90 in the training and validation set respectively] achieved relatively high performance than radiomics or clinics model

ROC analysis was performed to confirm the overall predictive performance of the radiomics, clinical and nomogram models according to the AUC. The AUCs of the radiomics, clinical and nomogram models in the training and validation sets are shown (Fig. 8). The AUCs of the clinical model were 0.85 (95% CI, 0.79–0.92) and 0.87 (95% CI, 0.79–0.95) in the training and validation sets, respectively. The selected radiomics feature-based radiomics model discriminated MSI status from MSS status, with AUCs of 0.81 (95% CI, 0.74–0.87) and 0.82 (95% CI, 0.72–0.92) in the training and validation sets, respectively. The nomogram model, which incorporated both the Rad-scores and clinical-CT reported information, showed superior predictive ability for MSI status than the other two models, with AUCs of 0.87 (95% CI, 0.81–0.93) and 0.90 (95% CI, 0.83–0.96) in the training and validation sets, respectively. The sensitivity, specificity, accuracy, negative predictive value (NPV), positive predictive value (PPV) and AUC of the three predictive models are summarized in Table 2.

**Table 2:** Comparison of the performance of the three models in predicting MSI status

Models	AUC	Sensitivity	Specificity	Accuracy	PPV	NPV
**Radiomics**						
**Training(*n*** **= 194)**	0.81 (0.74–0.87)	0.67	0.81	0.77	0.57	0.86
**Validation (*n*** **= 82)**	0.82 (0.72–0.92)	0.65	0.80	0.76	0.54	0.86
**Clinics**						
**Training(*n*** **= 194)**	0.85 (0.79–0.92)	0.77	0.88	0.84	0.70	0.89
**Validation (*n*** **= 82)**	0.87 (0.79–0.95)	0.79	0.80	0.79	0.59	0.90
**Nomogram**						
**Training(*n*** **= 194)**	0.87 (0.81–0.93)	0.82	0.83	0.82	0.65	0.91
**Validation (*n*** **= 82)**	0.90 (0.83–0.96)	0.80	1.00	0.80	1.00	0.73

*AUC* Area under curve, *PPV* Positive predictive value, *NPV* Negative predictive value

**Fig. 9:**
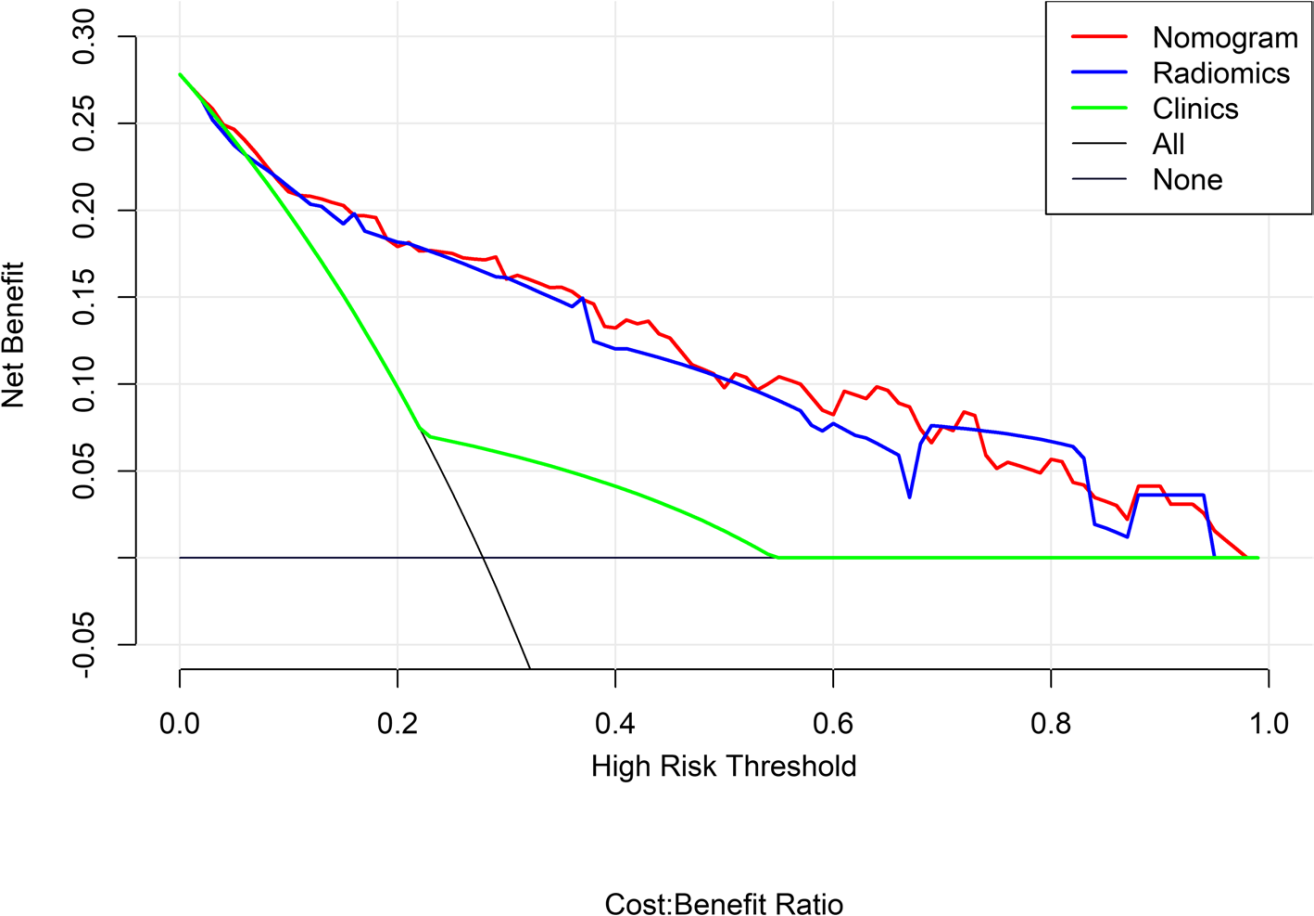
Decision curve analysis (DCA) of the radiomics model, clinics model and nomogram model. The X-axis is the threshold probability. The y-axis represents the net benefit, which is calculated by the difference between the expected benefit and the expected harm associated with the decision. The higher curve at a range threshold probability is the optimal prediction to maximize the net benefit. The decision curve shows that the nomogram model provides more net benefit than the other two models

Decision curve analysis (DCA) showed that the nomogram model obtained the highest net benefit compared with the other two models at a range threshold probability of 30–70%. The nomogram model achieved a net benefit similar to that of the radiomics model at ranges from 0 to 30% and 70 to 100% (Fig. 9).

## Discussion

In our retrospective study, we established a clinic-radiomics nomogram for individualized preoperative prediction of MSI status in CRC patients. Combining radiomics features from routine pretreatment portal venous phase CECT images with clinical factors and CT-reported status that are easily available in routine clinical practice, we achieved good predictive performance in both the training set (AUC, 0.87) and validation set (AUC, 0.90), with the incorporation of clinical risk factors, CT-reported status and radiomics features from routine pretreatment portal venous phase CECT images.

Our study showed an MSI prevalence of 18.83% in our CRC cases, which was consistent with the pathogenesis of merely 10–20% in previous studies [21–23]. In terms of clinical factors and CT-reported status of the CRC patients, our study demonstrated that location of primary tumor, WBC, CT reported IFS, histological grade were independent clinical risk factors and were closely associated with MSI status in CRC patients upon multivariable analysis. Among the four potential clinical risk factors, right-sided and poor differentiation were considered independent risk predictors closely associated with the MSI status of CRC patients, which is consistent with the findings of a previous study [24]. In our current study, we also found significant differences between the CT-reported IFS, WBC and MSI status. This interesting finding might be due to the inflammatory reaction accompanied by increased tumor-infiltrating lymphocytes and the presence of a peritumoral Crohn-like lymphocytic response in the MSI status of CRC patients [25, 26]. This finding was developed to initially identify MSI status cases preoperatively and to save costs. To the best of our knowledge, this might be the first study to explore the relationship between the CT-reported IFS, WBC and MSI status, and our result is recommended to further explorations based on larger samples and confirmed by further studies.

Recently, radiomics analysis has been a promising high-throughput method to extract a large number of nonvisible quantitative hidden features in medical images, which are related to intratumor heterogeneity. Radiomics analysis has been widely used in the field of routine clinical prognostic or treatment response evaluations, as well as survival prediction of CRC [10, 11, 27, 28]. In our current study, we extracted 1037 radiomics features from portal venous phase images, and twelve quantitative radiomics features were finally selected to calculate the Rad-score closely associated with the MSI status. Among them, 10 were texture features including LoG and wavelet transform GLCM, NGTDM, GLSZM and GLRLM, 1 was a first-order statistical feature and 1 was a shape feature. These 10 texture features provided a measure of nonuniformity of the gray levels in images, and thus, they were all recognized as parameters to reflect inherent intra-tumoral heterogeneity. Our study found that the Rad-score values of MSI CRC were significantly higher in CRC with MSS status, and we tried to explain the difference observed in this study. The higher Rad-score values in MSI CRC might be explained by the following: (1) a lower rate of cell proliferation activity and different cellular densities [29]; (2) the morphological characteristics of mixed mucinous, glandular and solid component leading to different cellular densities [26]; and (3) the increased tumor-infiltrating lymphocytes or presence of a peritumoral Crohn-like lymphocytic response [25], which all caused intratumor heterogeneity. Our results that the Rad-score values reflecting imaging heterogeneity were a pronounced biomarker for MSI CRC were basically consistent with previously published studies [13, 14, 30]. This finding demonstrated that the quantitative radiomics features of CRC might be of particular value in predicting MSI status and worthy of further study and exploration.

In the era of big data and precision medicine, a single clinical or radiomics model can no longer satisfy individualized prediction or diagnosis. Several studies [13, 14, 30, 31] have shown that the combination of clinical and radiomics models derived from primary CRC demonstrated considerable performance in the prediction of MSI status in CRC patients. The present study showed that the combined clinic and radiomic feature model had the highest AUC and showed superior discernibility in predicting the MSI status of CRC patients. However, we obtained a relatively higher AUC of 0.90 (0.83–0.96) than that in the abovementioned radiomics studies, except the study of Wu et al. [30]. This result might be attributed to the differences in patient cohorts, various scanners and the use of different analytical methods. Radiomics analysis may be regarded as the most promising comprehensive predictive approach to assist clinical practice and management. To promote clinical applications, we constructed a clinic-radiomics nomogram incorporating radiomics features and preoperative clinical features. The developing and validating nomogram could generate the probability of MSI status to achieve preoperative individualized prediction of MSI status in CRC patients by clinicians, consistent with the current trend of individualized and precision medicine.

However, our present study has several limitations. First, the retrospective nature of the study might lead to selection bias. Second, MSI status was assessed by a reliable IHC test, and PCR or next-generation sequencing (NGS) are recommended. Third, all sets of CT images used in our study were acquired from the same CT scanner. This fact might influence the generalization of our results. Fourth, the present study was only a single center with a limited sample size, and further validation is required in external and multicenter studies by using a larger sample size. The major limitation in our study was only a two-expert-controlled segmentation used to identify the gold standard, and some discrepancies caused by manually segmented ROIs were unavoidable. In the future, we will try to perform the true segmentation using the STAPLE algorithm as reported in the previous studies [32, 33].

## Conclusions

Our study presented and validated a radiomics-based nomogram that integrated radiomics features and clinical risk factors, which can be routinely used as a preoperatively individualized noninvasive and quantitative method for predicting MSI status in patients with CRC, assisting clinician personalized treatment decision-making, guiding personalized precision treatment and assessing patient prognosis in clinical practice.

## Supplementary Information

**Additional file 1.**

## Data Availability

All data generated or analyzed during this study are included in this published article.

## Abbreviations

CRC: Colorectal cancer
dMMR: Mismatch repair deficiency
MSI: Microsatellite instability
MSS: Microsatellite stability
CT: Computed tomography
CECT: Contrast-enhanced CT
CEA: Carcinoembryonic antigen
WBC: White blood cell count
TMS: Tumor maximum size
LN: Lymph node
IFS: Inflammatory response
ROIs: Regions of interest
ICC: Intra-and inter-class correlation coefficient
LoG: Laplacian of Gaussian
mRMR: Minimal-redundancy-maximal-relevance
LASSO: Least absolute shrinkage and selection operator
Rad-score: Radiomics signature score
ROC: Receiver operator characteristic
AUC: Area under the curve
NPV: Negative predictive value
PPV: Positive predictive value
DCA: Decision curves analysis
