# Supplementary material for: Development and validation of a radiomics-based nomogram for the preoperative prediction of microsatellite instability in colorectal cancer
Source: BMC Cancer. 2022 May 9;22:524. doi: 10.1186/s12885-022-09584-3 (PMC9087961; doi:10.1186/s12885-022-09584-3)
Supplement: Supplementary file 1 — Additional file 1. [file 12885_2022_9584_MOESM1_ESM.docx]

**Supplementary Table S1. Demographics comparison between training and validation datasets**

| **Characteristic** | **Level** | **Training (n=194)** | **Test (n=82)** | **p-value** |
| --- | --- | --- | --- | --- |
| **Age, mean (SD), years** |  | 63.9 (12) | 64.2 (13.3) | 0.830 |
| **gender, n (%)** | Female | 81 (41.8) | 41 (50.0) |  |
|  | Male | 113 (58.2) | 41 (50.0) | 0.259 |
| **location, n (%)** | Right colon | 43 (22.2) | 23 (28.0) |  |
|  | Left colon | 39 (20.1) | 12 (14.6) |  |
|  | Rectum | 112 (57.7) | 47 (57.3) | 0.412 |
| **CEA, n (%)** | Normal | 133 (68.6) | 57 (69.5) |  |
|  | Abnormal | 61 (31.4) | 25 (30.5) | 0.988 |
| **WBC, n (%)** | Normal | 161 (83.0) | 64 (78.0) |  |
|  | Abnormal | 33 (17.0) | 18 (22.0) | 0.425 |
| **CT-reported-TMS,**  **mean (SD), cm** |  | 4.6 (2.2) | 4.8 (2.1) | 0.641 |
| **CT-reported T stage, n (%)** | T1 | 6 (3.1) | 3 (3.7) |  |
|  | T2 | 11 (5.7) | 6 (7.3) |  |
|  | T3 | 55 (28.4) | 26 (31.7) |  |
|  | T4 | 122 (62.9) | 47 (57.3) | 0.843 |
| **CT-reported LN status, n (%)** | Negative | 128 (66.0) | 53 (64.6) |  |
|  | Positive | 66 (34.0) | 29 (35.4) | 0.939 |
| **CT-reported-IFR, n (%)** | No | 119 (61.3) | 53 (64.6) |  |
|  | Yes | 75 (38.7) | 29 (35.4) | 0.703 |
| **Histological grade, n (%)** | Well | 11 (5.7) | 4 (4.9) |  |
|  | Moderately | 152 (78.4) | 71 (86.6) |  |
|  | Poorly | 31 (16.0) | 7 (8.5) | 0.238 |
| **Rad-score (median [IQR])** |  | -0.5 [-1.3, 0.3] | -0.4 [-1.3, 0.2] | 0.835 |

CRC, colorectal cancer; CEA, carcinoembryonic antigen; WBC, white blood cell count; LN, lymph node; MSI, microsatellite instability; MSS, microsatellite stability. TMS, tumor maximum size; IFR, inflammatory response

**Supplementary Material S2**

Rad_score=-0.18*wavelet-HHL_ngtdm_Strength+-0.51*wavelet- HHL_glcm_Contrast+0.008*original_glszm_LargeAreaHighGrayLevelEmphasis+-0.02*log-sigma-2-0-mm-3D_ngtdm_Contrast+-0.308*original_shape_Sphericity+-0.252*original_firstorder_90Percentile+-0.2*wavelet-LHH_glrlm_ShortRunEmphasis+0.247*wavelet-HHH_ngtdm_Busyness+0.093*wavelet-LLH_firstorder_Energy+-0.049*wavelet-LHH_ngtdm_Strength+0.088*wavelet-LHL_glszm_SmallAreaEmphasis+0.128*wavelet-LHL_ngtdm_Busyness + -0.524
